# Using description logics to evaluate the consistency of drug-class membership relations in NDF-RT

**DOI:** 10.1186/s13326-015-0007-3

**Published:** 2015-03-28

**Authors:** Rainer Winnenburg, Jonathan M Mortensen, Olivier Bodenreider

**Affiliations:** Lister Hill National Center for Biomedical Communications, National Library of Medicine, National Institutes of Health, Bethesda, MD USA; Current Affiliation: Stanford Center for Biomedical Informatics Research, Stanford University, Stanford, CA 94305-5479 USA

**Keywords:** Ontology, Description logics, Quality assurance, National drug file-reference terminology

## Abstract

**Background:**

The NDF-RT (National Drug File Reference Terminology) is an ontology, which describes drugs and their properties and supports computerized physician order entry systems. NDF-RT’s classes are mostly specified using only necessary conditions and lack sufficient conditions, making its use limited until recently, when asserted drug-class relations were added. The addition of these asserted drug-class relations presents an opportunity to compare them with drug-class relations that can be inferred using the properties of drugs and drug classes in NDF-RT.

**Methods:**

We enriched NDF-RT’s drug-classes with sufficient conditions, added property equivalences, and then used an OWL reasoner to infer drug-class membership relations. We compared the inferred class relations to the recently added asserted relations derived from FDA Structured Product Labels.

**Results:**

The inferred and asserted relations only match in about 50% of the cases, due to incompleteness of the drug descriptions and quality issues in the class definitions.

**Conclusions:**

This investigation quantifies and categorizes the disparities between asserted and inferred drug-class relations and illustrates issues with class definitions and drug descriptions. In addition, it serves as an example of the benefits DL can add to ontology development and evaluation.

**Electronic supplementary material:**

The online version of this article (doi:10.1186/s13326-015-0007-3) contains supplementary material, which is available to authorized users.

## Introduction

We rely on ontologies throughout biomedicine, from the life sciences to the clinic [1]. As Electronic Health Record adoption increases in the clinic, so too will the reliance on the ontologies that facilitate their meaningful use. Clinical decision support and analytics are functions supported by ontologies. For example, computerized physician order entry (CPOE) systems typically leverage drug ontologies to ensure that patients are safely prescribed drugs in accordance with clinical guidelines (e.g., [2]).

An example of such an ontology is the National Drug File-Reference Terminology (NDF-RT), an extension to the drug formulary used by the Veterans Administration and developed using a description logics (DL) formalism. It provides a rich description of pharmacologic classes in reference to properties, such as mechanism of action, physiologic effect, chemical structure and therapeutic intent. NDF-RT can be leveraged to prevent a patient allergic to penicillin drugs from being prescribed amoxicillin, a penicillin antibacterial.

However, NDF-RT only specifies *necessary* conditions for class membership to the pharmacologic classes, but not *sufficient* conditions. (In DL parlance, these classes are “primitive”, not defined.) As a consequence, a DL reasoner is unable to classify automatically drugs as members of a given pharmacologic class, even when both drugs and pharmacologic classes are described in terms of the same properties. The inability to classify drugs into their classes limits the usefulness of NDF-RT in systems like CPOE that rely on such information.

In previous work, where we overcame this limitation by augmenting the pharmacologic classes with necessary and sufficient conditions, we found that we could infer drug-class membership relations effectively [3]. Specifically, we demonstrated the use of a modified version of NDF-RT for clinical decision purposes (patient classification). One limitation of this work was that we did not evaluate the inferred drug-class membership relations beyond our proof-of-concept application.

NDF-RT recently integrated authoritative drug-class membership assertions extracted from the Structured Product Labels (package inserts) by the Food and Drug Administration (FDA), along with a specification of the drugs in terms of the same properties used for specifying the classes. These assertions remove the drug-class membership limitation we highlighted earlier, instead providing explicit drug-class membership relations that do not rely on DL reasoning. But precisely because these asserted drug-class relations have been made independently of the logical definitions of the classes, there is the possibility for the asserted and inferred drug-class membership relations to be inconsistent.

The objective of this work is to evaluate the consistency of the drug-class membership relations that were inferred from the pharmacologic class definitions and drug descriptions, against the newly asserted, authoritative drug-class membership relations. This evaluation is also an indirect contribution to the assessment of the class definitions and the drug descriptions in terms of completeness and consistency (i.e., agreement between information sources).

## Background

### NDF-RT drugs and classes

The National Drug File Reference Terminology (NDF-RT) is a resource developed by the Department of Veterans Affairs (VA), Veterans Health Administration, as an extension of the VA National Drug File [4]. Like other modern biomedical terminologies, NDF-RT is developed using description logics and is available in native XML format. The version used in this study is the latest version available, dated November 3, 2014, downloaded from [5], from which we derived our augmented representation.

This version covers 7,287 active moieties (DRUG_KIND, level = ingredient), as well as 543 Established Pharmacologic Classes (EPCs) specified in reference to some of the properties of the active moieties. NDF-RT now contains several sources of relations between drugs and their properties. The April 2014 version of NDF-RT introduced a new set of relations between drugs and their properties originating from the class indexing file released as part of DailyMed, identified by the suffix “FDASPL”. Moreover, this version also introduced authoritative drug-class membership assertions from the same source. Finally, NDF-RT also provides a specification of the EPCs in reference to the same properties used for describing the drugs themselves, provided by “Federal Medication Terminologies subject matter experts” and identified by the suffix “FMTSME”. In this work, we focus on the drug-property assertions from FDASPL, class-property assertions from FMTSME, and drug-class assertions provided by the FDA.

### Description logics

In short, Description Logics (DL) are a set of logical constructs with which one can develop ontologies. Krötzsch and colleagues provide a more formal introduction to DL [6]. Like other knowledge representation methods, DL allows one to specify, in a computable fashion, the entities (i.e., *classes*) that exist in a given domain and the relationships (i.e., *relations*) between them. In comparison to older methods of knowledge representation, DL ensures common, unambiguous semantics so that the ontology’s interpretation is consistent across software and users. This consistent logical underpinning enables the use of reasoners, which are programs that compute (i.e., infer) the logical entailments (i.e., conclusions) of a given ontology. For example, if *Alprostadil has physiologic effect Venous dilation* and *Venous dilation is-a Vasodilation*, a reasoner concludes that *Alprostadil has physiologic effect Vasodilation*. A typical approach to developing ontologies with DL is to specify a set of properties that each class has (e.g., *Penicillin antibacterial has ingredient Penicillin* and *treats or prevents Bacterial infection*; *Antiseptic treats or prevents Bacterial infection*) and then infer the additional relations among classes. With a set of specified classes, a reasoner can then classify them into an inferred hierarchy. In our example, the inferred hierarchy would show that *Penicillin antibacterial is-a Antiseptic*. In the context of this study, NDF-RT uses this same approach, specifying EPCs in terms of their properties. Unlike the example above, however, pharmacologic classes in NDF-RT (EPCs) are “primitive”, in that they only specify the *necessary* conditions of class membership, and therefore prevent a reasoner from constructing a useful inferred hierarchy. Later, we describe how we enrich NDF-RT with *sufficient* conditions so that we can take full advantage of a reasoner.

In this work, we use OWL, the web ontology language, a web standard for developing ontologies that leverages DL. OWL is the *de facto* standard for biomedical ontologies and there is a suite of tools for developing OWL ontologies, including development environments such as Protégé [7] and reasoners such as HermiT [8].

### Related work

In addition to being used as a framework for building ontologies, DL has been shown to be useful for reasoning with biomedical entities, including protein phosphatases [9] and penetrating injuries [10]. However, to our knowledge, DL reasoning has not yet been applied to the automatic classification of drugs, except for our previous work on anti-coagulants [3].

NDF-RT is used frequently as a resource for standardizing pharmacologic classes (e.g., [11,12]). However, investigators generally use the drug properties as classes (e.g., drugs that have the physiologic effect “decreased coagulation activity” for anti-coagulants), rather than the EPCs. Moreover, only asserted relations are used in most investigations, as opposed to inferred drug-class relations.

The specific contribution of this paper is the augmentation of the logical definitions of pharmacologic classes in NDF-RT to enable the automatic inference of drug-class membership relations using a DL reasoner. We substantially extend our previous work on anticoagulants, by generalizing it to all pharmacologic classes and providing a comparison to authoritative, asserted drug-class relations from the FDA.

## Methods

Our approach to evaluating inferred drug-class membership relations in NDF-RT is summarized as follows. First, we converted the NDF-RT data from their original format (XML) to a DL format (OWL). This conversion process augments the EPCs with necessary and sufficient conditions. These conditions allowed a DL reasoner to classify drugs into their respective classes using the class definitions and the properties of drugs. We created two OWL datasets. One, used as a gold standard, only contains the asserted, authoritative drug-class relations. In contrast, these asserted relations have been removed from the second dataset, so that only inferred drug-class relations were present after the reasoner runs (i.e., inferred by the reasoner). We ran a DL reasoner and then compared inferred and asserted drug-class relations from the perspective of drugs and from that of classes.

In order to restrict this investigation to clinically significant drugs, we mapped all NDF-RT ingredients to RxNorm and required that ingredients be linked to clinical drugs. We further normalized all ingredients to base ingredients in RxNorm, to abstract away from minor differences in ingredients, including salts, esters and complexes, which rarely affect drug-class membership. In practice, we mapped the “precise ingredients” in RxNorm (e.g., *albuterol sulfate*) to their base ingredient (*albuterol*). Multi-ingredient drugs were ignored, because there is often more variability in their classification.

### Augmenting pharmacologic classes with sufficient conditions

In order to produce the two OWL datasets used for comparing asserted and inferred drug-class relations, we started by creating a “baseline” OWL representation from the original XML dataset, which we used as our asserted dataset (dataset “A”). Next, as previously described in [3], we transformed the primitive EPCs into defined classes by taking the existing set of properties for each class (i.e., necessary conditions) and using them to “define” the class. In particular, all properties are folded into a single owl:equivalentClass (≡) axiom, thereby specifying necessary and sufficient conditions of each class. For the purpose of this work, we focus on the three main properties used for the description of the drugs (mechanism of action, physiologic effect and chemical structure). Additionally, we leveraged the therapeutic intent relations (may_treat and may_prevent) present in NDF-RT, because many EPCs refer to them in their definitions. These relations link drugs and EPCs to disease entities.

We further modified this OWL file by applying a series of transformations that are necessary for enabling proper inference (dataset “I”). We harmonized the names of roles used in the definition of the classes (e.g., *has_MoA_FMTSME*) with those used in the description of the drugs (e.g., *has_MoA_FDASPL*) by creating owl:equivalentProperty axioms between them. The following equivalences are created:

- *has_MoA_FMTSME* ≡ *has_MoA_FDASPL* (for mechanism of action),
- *has_PE_FMTSME* ≡ *has_PE_FDASPL* (for physiologic effect),
- *has_Chemical_Structure_FMTSME* ≡ *has_Chemical_Structure_FDASPL*,
- *may_treat_FMTSME* ≡ *may_treat_NDFRT*, and
- *may_prevent_FMTSME* ≡ *may_prevent_NDFRT*.

### Inferring relations between drugs and EPCs

**Figure 1:**
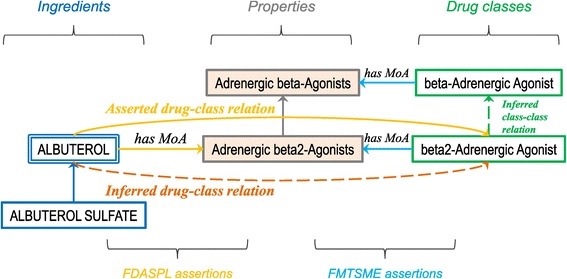
**Method overview.** Relations between the drug *albuterol* and the class *beta2-Adrenergic Agonist [EPC]*, with asserted and inferred drug-class relations. Note that there is only one direct path from ingredients to pharmacologic classes through the recently added yellow asserted drug-class relation. In this study, we compare how often inference using the properties, which produces the dashed orange line, recapitulates the solid yellow line.

Next, we leveraged an OWL reasoner to infer the drug-class membership relations from the class definitions and the descriptions of drugs. Using the necessary and sufficient conditions we created for the classes, an OWL reasoner infers a subclass relation between a drug and a pharmacologic class when the properties of the drug and those of the pharmacologic class are shared. For example, the class *beta2-Adrenergic Agonist [EPC]* (*N0000175779*) is defined as equivalent to (*'Pharmaceutical Preparations' and (has_MoA_FMTSME some 'Adrenergic beta2-Agonists [MoA]')*). The drug *albuterol* (*N0000147099*) has the property *has_MoA_FDASPL some 'Adrenergic beta2-Agonists [MoA]'*, and is therefore inferred as being a subclass of *beta2-Adrenergic Agonist [EPC]*. (The inference will also occur if the property of the drug is a subclass of the property used in the definition of the class). Figure 1 provides a schematic of the above example.

A secondary benefit of the classification with an OWL reasoner is that it creates a hierarchy of the pharmacologic classes themselves, based on their logical definitions. For example, *beta2-Adrenergic Agonist [EPC]* (*N0000175779*) is inferred to be a subclass of *beta-Adrenergic Agonist [EPC]* (*N0000175555*), because the definition of *beta2-Adrenergic Agonist [EPC]* shown earlier is more specific than that of *beta-Adrenergic Agonist [EPC]* (*'Pharmaceutical Preparations' and (has_MoA_FMTSME some 'Adrenergic beta-Agonists [MoA]')*). For this reason, we reclassified both OWL datasets, although no inferred drug-class relations were generated in dataset “A”.

**Figure 2:**
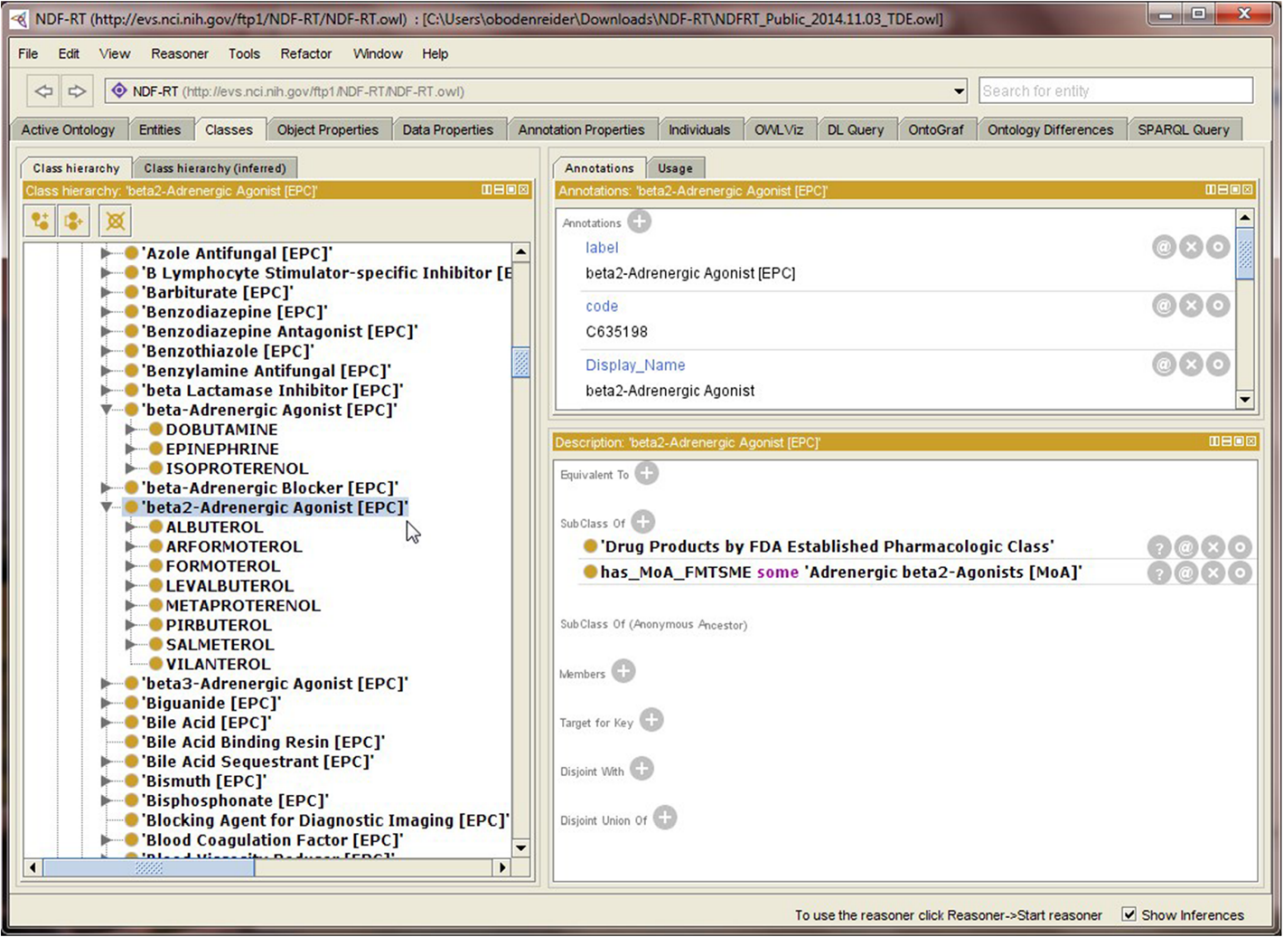
**Primitive class** ***Adrenergic Decongestant [EPC].*** *beta2-Adrenergic Agonist [EPC]* appears as a primitive class in the default distribution of NDF-RT.

**Figure 3:**
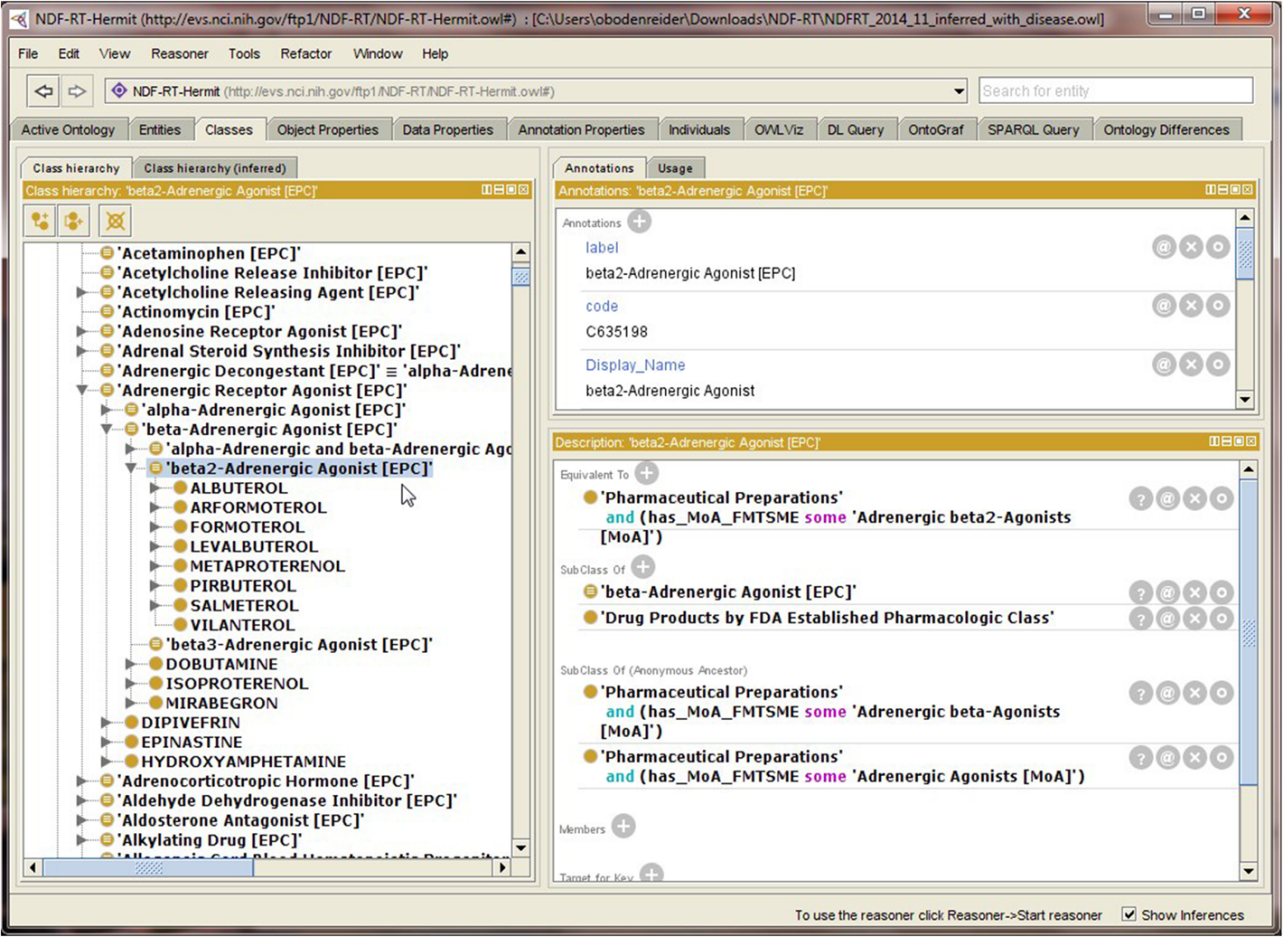
**Defined class** ***Adrenergic Decongestant [EPC].*** The appearance of *beta2-Adrenergic Agonist [EPC]*in Protégé after augmenting it with sufficient conditions.

Figure 2 provides a screenshot from Protégé of a pharmacologic class before enrichment and Figure 3 shows its definition after. Before enrichment, the class *beta2-Adrenergic Agonist [EPC]* has no sufficient conditions (the section “Equivalent To” is empty) and the EPCs are not hierarchically related (*beta2-Adrenergic Agonist [EPC]* and *beta-Adrenergic Agonist [EPC]* are at the same hierarchical level, i.e., part of a flat list of EPCs). The drug *albuterol* is asserted to be a member of the class *beta2-Adrenergic Agonist [EPC]*. In contrast, after enrichment (and reclassification), the class *beta2-Adrenergic Agonist [EPC]* has acquired sufficient conditions (visible in the section “Equivalent To”) and the EPCs are now hierarchically related (*beta2-Adrenergic Agonist [EPC]* is a subclass of *beta-Adrenergic Agonist [EPC]*). The drug *albuterol* is inferred to be a member of the class *beta2-Adrenergic Agonist [EPC]*.

### Comparing asserted and inferred drug-class relations

We compared asserted (dataset “A”) and inferred (dataset “I”) drug-class relations from the perspective of drugs and pharmacologic classes, respectively. In both cases, we issued queries against the OWL datasets (after reclassification). For each drug, we queried its set of pharmacologic classes in each dataset and determined which classes are common to both datasets vs. specific to one dataset. For example, the drug *albuterol* (*N0000147099*) has the same class in both datasets, *beta2-Adrenergic Agonist [EPC]* (*N0000175779*). In contrast, the drug *hydrochlorothiazide* (*N0000145995*) has an asserted relation to *Thiazide Diuretic [EPC]* (*N0000175419*), but an inferred relation to *Thiazide-like Diuretic [EPC]* (*N0000175420*). For each pharmacologic class, we queried its set of drugs in each dataset and determined which drugs are common to both datasets vs. specific to one dataset. In order to consider higher-level classes to which drugs are not direct members, we used the transitive closure of the hierarchical relation rdfs:subClassOf. As a consequence, a given class will have as members not only its direct drugs, but also the members of all its subclasses. For example, in both the “A” and “I” datasets, the class *beta-Adrenergic Agonist [EPC]* has the base ingredient *albuterol* as an indirect member through its subclass class *beta2-Adrenergic Agonist [EPC]*. Of note, the salt ingredient *albuterol sulfate* is ignored as a result of the normalization to RxNorm base ingredients described earlier.

### Implementation

The modifications described above were performed using an XSL (eXtensible Stylesheet Language) transformation. The resulting OWL file was classified with HermiT 1.2.2 [8]. Protégé 5.0 was used for visualization purposes [7]. The OWL file containing the inferences computed by the reasoner was loaded in the open source triple store Virtuoso 7.10 [13]. The query language SPARQL was used for querying drug-class relations

## Results

### Asserted and inferred drug-class relations

#### Drugs

Of the 7,352 drugs (at the ingredient level) in NDF-RT, 3,351 are identifiable as clinically relevant ingredients in RxNorm. After normalization to base ingredients, 2,247 drugs remain, of which 1,308 have at least one relation to a pharmacologic class (EPC). As shown in Table 1, all but 48 drugs (1,260) have asserted drug-class relations and 1,011 drugs have inferred relations. 963 drugs have both asserted and inferred relations.

**Table 1:** **Drug-class relations (direct), drug perspective**

**Drugs related to drug classes**	**#**	**%**
Drugs with identical sets of classes for the asserted and inferred drug-class relations	660	50.46
Drugs with compatible sets of classes (each class from the asserted is identical to or hierarchically related to a class in the inferred set)	127	9.71
Drugs with additional drug-class relations in the asserted set only	68	5.20
Drugs with additional drug-class relations in the inferred set only	73	5.58
Drugs with additional drug-class relations in both the asserted and inferred set	35	2.68
Drugs with asserted drug-class relations only (no inferred relations)	297	22.71
Drugs with inferred drug-class relations only (no asserted relations)	48	3.67
**Total number of related drugs**	**1308**	**100.00**

#### Pharmacologic classes

Of the 553 pharmacologic classes (EPC) in NDF-RT, 463 have relations to drugs, of which all but five (458) have asserted relations and 340 have inferred relations (as shown in Table 2). In total, 335 of the 463 classes have both asserted and inferred relations to drugs.

**Table 2:** **Drug-class relations (direct and indirect), class perspective**

**Drug classes related to drugs**	**#**	**%**
Classes with identical sets of drugs for the asserted and inferred drug-class relations	**242**	52.27
Classes with additional drug-class relations in the asserted set only	**55**	11.88
Classes with additional drug-class relations in the inferred set only	**20**	4.32
Classes with additional drug-class relations in both the asserted and inferred set	**18**	3.89
Classes with asserted drug-class relations only (no inferred relations)	**123**	26.57
Classes with inferred drug-class relations only (no asserted relations)	**5**	1.08
**Total number of related classes**	**463**	**100.00**

#### Drug-class relations

**Figure 4:**
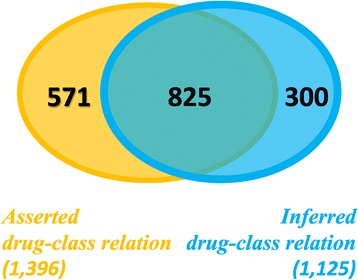
**Comparison of asserted and inferred classes.** 59% of the 1,396 asserted classes are also inferred and 77% of the 1,125 inferred classes are also asserted.

As shown in Figure 4, there are 1,396 asserted and 1,125 inferred direct drug-class relations, of which 825 (59% and 77%, respectively) are in common. Of the asserted relations, 571 (41%) could not be inferred, whereas 300 (27%) inferred relations are not present in the asserted set. Considering the transitive closure of the hierarchical relation rdfs:subClassOf (for the drug class perspective), we obtain 2,211 asserted and 1,513 inferred drug-class relations, of which 1,332 (40% and 88%, respectively) are in common. Of the asserted relations 879 (40%) could not be inferred, whereas 181 (12%) inferred relations are not present in the asserted set.

### Perspective of drugs

For each drug, we compare the set of (direct) pharmacologic classes in datasets “A” and “I”. The various types of differences observed between asserted and inferred drug-class relations are presented in Table 1. The largest category corresponds to drugs with identical sets of asserted and inferred drug-class relations (50%). For example, the drug *imatinib* has the same class *Kinase Inhibitor [EPC]* in both datasets. Drugs with asserted drug-class relations, but lacking inferred drug-class relations represent 23% of the cases. For example, the drug *losartan* has the class *Angiotensin 2 Receptor Blocker [EPC]* in dataset “A”, but no class in dataset “I”.

### Perspective of pharmacologic classes

For each pharmacologic class, we compare the set of (direct and indirect) drug members in datasets “A” and “I”. The various types of differences observed between asserted and inferred drug-class relations are presented in Table 2. As we observed for drugs, the largest category corresponds to EPCs with identical sets of asserted and inferred drug-class relations (52%). For example, the class *Monoamine Oxidase Inhibitor [EPC]* has the same five drugs in both datasets, including *isocarboxazid* and *rasagiline*. EPCs with asserted drug-class relations, but lacking inferred drug-class relations also represent about 27% of the cases. For example, the class *Quinolone Antibacterial [EPC]* has eight drugs in dataset “A”, including *ofloxacin* and *levofloxacin*, but no members in dataset “I”.

## Discussion

### Disparities between asserted and inferred drug-class relations

#### Missing inferences

As mentioned in the results, the largest category of disparity is represented by missing inferred drug-class relations, including cases where there are no inferred relations at all and cases where inferred relations only cover part of the asserted relations. Missing inferences should not be interpreted as an inherent failure of the OWL reasoner to identify drug-class relations, but rather as issues with the completeness and quality of class definitions and drug descriptions (see below for details). For example, the reason why the drug *lurasidone*, a drug indicated for the treatment of schizophrenia, has an asserted, but not inferred drug-class relation to *Atypical Antipsychotic [EPC]* is because the therapeutic intent of *lurasidone* (*Schizophrenia and Disorders with Psychotic Features*) is not described in the dataset. In fact, there is no drug property asserted for *lurasidone* by FDASPL. Another example is the drug *ofloxacin* mentioned earlier. In this case, the asserted EPC (*Quinolone Antimicrobial [EPC]*) is not inferred because its definition includes both *may_treat Infectious Diseases* and *may_prevent Infectious Diseases*, while the drug description only includes treatment, not prevention (e.g., *may_treat 'Klebsiella Infections*). Similarly, the description of the drug *ipilimumab* is too underspecified to match the definition of its asserted class, *CTLA-4-directed Blocking Antibody [EPC].* In addition to *has_MoA CTLA-4-directed Antibody Interactions*, which is in the drug description, the EPC also makes references to the physiologic effect (*has_PE Increased Immunologic Activity* and *has_PE Increased T Lymphocyte Activation*).

#### Inferences with no corresponding asserted relations

The number of cases (156 drugs and 43 classes) where inferred drug-class relations are found when there is no asserted drug-class relation (or a different asserted drug-class relation) is interesting as it can help detect potentially missing asserted relations. For example, the drug *bupropion* has a single asserted relation to the structural class *Aminoketone [EPC]*. However, it has an inferred relation to *Norepinephrine Reuptake Inhibitor [EPC]* (through its mechanism of action, *Norepinephrine Uptake Inhibitors [MoA]*). In this case, the set of asserted relations, which we use as our reference, seems to be incomplete. Another example is the drug *isosorbide*, an anti-angina agent, for which we correctly infer the class *Anti-anginal [EPC]*, while no asserted EPC is present. Here again, the reference is incomplete.

#### Inconsistent drug-class relations due to granularity differences

Drug-class relations from dataset “A” tend to associate drugs with more specific classes than in dataset “I”. For example, the antibiotic *amikacin* is associated with *Aminoglycoside Antibacterial [EPC]* (through asserted relations), but with the less specific *Aminoglycoside [EPC]* (through inferred relations). The reason here is similar to what was described earlier for the antibiotic *ofloxacin*, i.e., discrepancy between *may_treat* and *may_prevent* vs. only *may_treat* properties on the side of the EPC and the drug, respectively. As shown in Table 1, we identified 127 drugs for which the classes in sets “A” and “I” are hierarchically related. Of these, there are only 4 cases with an inferred relation to a class that is more specific than the class involved in the asserted relation.

### Specific contribution of the therapeutic intent relations

The DailyMed indexing file provided by the FDA (FDASPL) only contains drug descriptions in reference to mechanism of action, physiologic effect and chemical structure, not therapeutic intent. However, many EPC definitions refer to *may_treat* and *may_prevent* relations. Therefore, no drug-class relations to these classes can be inferred, because the corresponding relations are missing from the drug descriptions. Therapeutic intent relations are available for the drugs as part of the set of legacy relations provided by NDF-RT (not FDASPL). We used these relations to complement the relations from FDASPL in order to maximize our chances to infer drug-class relations to the EPCs. We assessed the specific contribution of the therapeutic intent relations to the inference of drug-class relations by computing a “baseline” without using the therapeutic intent relations and comparing it to our dataset “I”.

As shown in Table 3, the use of therapeutic intent relations (column “+DISEASE”) allows us to infer drug-class relations for an additional 46 drugs compared to the baseline. There are fewer drugs (82) for which we only have asserted drug-class relations. Surprisingly, however, the number of drugs for which the asserted and inferred classes are the same has not significantly increased, which indicates that the drug-class relations inferred with the use of therapeutic intent tends to be different from the asserted drug-class relations.

**Table 3:** **Specific contributions of enhancement step**

**Drug perspective**	**Baseline**		**+ Disease**		**Delta**
**# drugs with**	**Abs.**	**%**	**Abs.**	**%**	
**Identical sets**	657	52.06	660	50.46	3
**Compatible sets**	122	9.67	127	9.71	5
**Additional asserted**	66	5.23	68	5.20	2
**Additional inferred**	18	1.43	73	5.58	55
**Additional in both**	18	1.43	35	2.68	17
**Asserted only**	379	30.03	297	22.71	−82
**Inferred only**	2	0.16	48	3.67	46
**Total # drugs**	**1262**	**100**	**1308**	**100**	46
**Total # pairs**	**1569**		**1696**		127
**Drug class perspective**	**Baseline**		**+ Disease**		**Delta**
**# classes with**	**Abs.**	**%**	**Abs.**	**%**	
**Identical sets**	237	51.19	242	52.27	5
**Additional asserted**	40	8.64	55	11.88	15
**Additional inferred**	19	4.10	20	4.32	1
**Additional in both**	5	1.08	18	3.89	13
**Asserted only**	157	33.91	123	26.57	−34
**Inferred only**	5	1.08	5	1.08	0
**Total # classes**	**463**	**100**	**463**	**100**	0
**Total # pairs**	**2259**		**2392**		133

For example, the drug *citalopram* was only associated with the inferred class *Serotonin Reuptake Inhibitor [EPC]* in the baseline (based on its mechanism of action), which was also its asserted EPC. In addition, it acquires a relation to *Mood Stabilizer [EPC]* when using the therapeutic intent relations, resulting in one additional inferred class compared to the asserted class. This example illustrates why the use of therapeutic intent relations does not significantly increase the number of drugs with similar sets of asserted and inferred classes.

### Description logics and quality assurance

There is a range of automated ontology quality assurance methods in the literature [14]. The results of this work highlight the usefulness of DL for that task. Here, we enriched the logic in NDF-RT to enable us to evaluate the quality and completeness of new, explicitly-added knowledge. Indeed, such rich logic allows for a quick evaluation at minimal cost. In this work, we had a reference against which to compare. However, when a gold standard is not available, DL reasoners can still check consistency and satisfiability, automatically detecting logical contradictions that usually indicate an error exists in the ontology. For instance, Horridge et al. used reasoning to identify contradictions within ICD-11 [15]. Unfortunately, even considering the benefits of a richly defined ontology, Noy and colleagues confirmed empirically that most biomedical ontologies do not use rich semantics but instead rely mostly on simple hierarchical subsumption relations [16].

## Conclusions

As we rely increasingly on ontologies, it is important to ensure their content is complete and correct. In this work, we developed a methodology to evaluate the content of NDF-RT using description logics. We found that the inferred and asserted relations only matched in about 50% of the cases. Ideally, the asserted and inferred drug-class relations should be identical. Our results suggest that there is an opportunity for quality assurance of NDF-RT content (completeness of the drug descriptions and quality of the class definitions). This work serves as an exemplar of how DL can enhance ontology development and evaluation and shows ontology developers that a little semantics can go a long way.

## Abbreviations

NDF-RT: National drug file – reference terminology
XML: Extensible markup language
VA: Veterans affairs
EPC: Established pharmacologic classes
XSL: Extensible stylesheet language
DL: Description logics
OWL: Web ontology language
ATC: Anatomic therapeutic chemical classification system
FDA: Food and drug administration
SPARQL: SPARQL Protocol and RDF query language
ICD-11: International classification of diseases 11th revision
